# A Qualitative Exploration of Nutrition Screening, Assessment and Oral Support Used in Patients Undergoing Cancer Surgery in Low- and Middle-Income Countries

**DOI:** 10.3390/nu14040863

**Published:** 2022-02-18

**Authors:** Anne Marie Sowerbutts, Stephen R. Knight, Marie Carmela M. Lapitan, Ahmad U. Qureshi, Mayaba Maimbo, Edwin Mwintiereh Ta-ang Yenli, Stephen Tabiri, Dhruva Ghosh, Pamela Alice Kingsley, Sudha Sundar, Catherine A. Shaw, Apple Valparaiso, Cristina Almira Alviz, Aneel Bhangu, Evropi Theodoratou, Thomas G. Weiser, Ewen M. Harrison, Sorrel T. Burden

**Affiliations:** 1 Faculty of Medicine, Biology and Health, and Manchester Academic Health Science Centre, University of Manchester, Oxford Road, Manchester M13 9PL, UK; annemarie.sowerbutts@manchester.ac.uk; 2 NIHR Global Research Unit on Global Surgery, Centre for Medical Informatics, Usher Institute, University of Edinburgh, Edinburgh EH16 4SB, UK; stephenknight@doctors.org.uk (S.R.K.); catherine.shaw@ed.ac.uk (C.A.S.); mail@ewenharrison.com (E.M.H.); 3 Institute of Clinical Epidemiology, National Institutes of Health and Department of Surgery, University of the Philippines, Manila 1101, Philippines; mmlapitan@up.edu.ph; 4 Department of Surgery, King Edward Medical University, Lahore 54000, Pakistan; ahmeduzairq@gmail.com; 5 Department of General Surgery, Kitwe Teaching Hospital, Kitwe 10101, Zambia; mayaba_m@yahoo.co.uk; 6 Department of Surgery, School of Medicine, University for Development Studies, Tamale 00233, Ghana; mwintyus@yahoo.com (E.M.T.-a.Y.); kstephenba14@gmail.com (S.T.); 7 Department of Paediatric Surgery, Christian Medical College, Ludhiana 141008, India; dhruvghosh73@gmail.com; 8 Department of Radiation Oncology, Christian Medical College, Ludhiana 141008, India; pamelajeyaraj@yahoo.co.in; 9 Institute of Cancer and Genomic Sciences, University of Birmingham, Birmingham B15 2SQ, UK; s.s.sundar@bham.ac.uk; 10 Department of Surgery, Philippine General Hospital, Manila 1101, Philippines; apvalparaisomd@gmail.com (A.V.); cristinaalmira30@gmail.com (C.A.A.); 11 NIHR Global Research Unit on Global Surgery, Institute of Translational Medicine, Heritage Building, Mindelsohn Way, Birmingham B15 2TH, UK; a.a.bhangu@bham.ac.uk; 12 Centre for Global Health, Usher Institute, University of Edinburgh, Edinburgh EH8 9AG, UK; e.theodoratou@ed.ac.uk; 13 Department of Clinical Surgery, University of Edinburgh, Edinburgh EH16 4SA, UK; thomas.weiser@ed.ac.uk; 14 Department of Surgery, Stanford University, Stanford, CA 94305, USA; 15 Intestinal Failure Unit, Salford Royal NHS Foundation Trust, Manchester M6 8HD, UK

**Keywords:** cancer, low- and middle-income countries, oncology, perioperative, surgery, undernutrition

## Abstract

Preoperative undernutrition is a prognostic indicator for postoperative mortality and morbidity. Evidence suggests that treating undernutrition can improve surgical outcomes. This study explored the provision of nutritional screening, assessment and support on surgical cancer wards in low- and middle-income countries (LMICs). This was a qualitative study and participants took part in one focus group or one individual interview. Data were analysed thematically. There were 34 participants from Ghana, India, the Philippines and Zambia: 24 healthcare professionals (HCPs) and 10 patients. Results showed that knowledge levels and enthusiasm were high in HCPs. Barriers to adequate nutritional support were a lack of provision of ward and kitchen equipment, food and sustainable nutritional supplements. There was variation across countries towards nutritional screening and assessment which seemed to be driven by resources. Many hospitals where resources were scarce focused on the care of individual patients in favour of an integrated systems approach to identify and manage undernutrition. In conclusion, there is scope to improve the efficiency of nutritional management of surgical cancer patients in LMICs through the integration of nutrition assessment and support into routine hospital policies and procedures, moving from case management undertaken by interested personnel to a system-based approach including the whole multidisciplinary team.

## 1. Introduction

There are five billion people who do not have access to safe, affordable and timely surgery, and only 6% of all operations performed worldwide are conducted in low- and middle-income countries (LMICs) [1]. For cancer, curative treatment for the majority of early-stage solid tumours is achieved through surgical resection [2]. Surgery in LMICs is often unavailable or outside the economic reach of the majority of the population, often resulting in catastrophic healthcare expenditure [3]. 

One in every nine people worldwide is undernourished, and in 2019 there were 690 million people who were hungry [4], which increased from previous years due to the coronavirus pandemic and higher levels of food insecurity [5]. There is a high prevalence of undernutrition in LMICs, with the poorest countries having higher levels of undernutrition within their populations [5]. Recently, undernutrition when measured using the Global Leadership Initiative on Malnutrition (GLIM) criteria was reported to be disproportionately represented in gastric and colorectal cancer patients undergoing elective surgery in LMICs compared to high-income countries [6]. Undernutrition was found to be an independent risk factor for death or major complications within 30 days of surgery [6]. 

Undernutrition is one of the few modifiable risk factors contributing to higher surgical mortality and is a negative prognostic factor for morbidity and mortality in surgical patients [7]. When nutritional interventions are given to patients undergoing cancer surgery, a reduction in percentage weight loss and increased nutritional intake has been demonstrated [8]. Subsequently, the use of preoperative oral nutrition supplements has been linked to a reduction in postoperative morbidity in colorectal cancer patients and mixed surgical groups [9,10,11]. Hence, evidence has shown that undernutrition is treatable in surgical cancer patients in the absence of refractory cachexia [12]

The Global Surgery 2030 Roadmap recommended improving access to timely and essential surgery and decreasing perioperative mortality against their international targets to improve surgical outcomes [1]. Unsurprisingly, given the association between undernutrition and postoperative mortality, nutrition was identified as a priority area for research at an international annual prioritisation meeting involving surgeons from LMICs [13]. Oral nutritional interventions are easy to administer and economically viable. However, before proposing research to improve preoperative nutrition in LMICs, a better understanding of the landscape of nutritional services, together with an exploration of barriers and facilitators of good clinical practice, is required. 

This exploratory investigation aims to establish baseline knowledge of nutritional screening, assessment and nutritional support interventions used on surgical cancer wards in LMICs for the identification and treatment of undernutrition. Secondary aims were to understand the organisation and management of nutritional services in LMICs along with barriers and facilitators to implementing optimum nutritional practice within clinical environments in LMICs.

## 2. Materials and Methods

### 2.1. Participants

Qualitative semi-structured interviews were conducted one-on-one with healthcare professionals (HCPs) and patients on surgical cancer wards, together with a focus group with HCPs. Inclusion criteria were any HCPs working in a hospital with surgical cancer patients or any patient on a surgical cancer ward in a LMIC defined by the Organisation for Economic Co-operation and Development (OECD) and the Development Assistance Committee (DAC) [14]. A snowballing technique was used to obtain a sample of HCPs, with interviewees recommending colleagues with an interest in nutritional and operational procedures at the ward level who worked with cancer patients [15]. Potential participants were approached either face-to-face or by email. Appointments were made with HCPs for the interviews at a surgical meeting held in Ghana in 2019 or by researchers working in either Ghana, the Philippines or India. For patients, a convenience sample was used. Patients were asked to participate by the interviewer if they were an inpatient between January and March 2021.

The sample was chosen to reflect different LMICs and the patient sample was included to provide triangulation of data [16]. As the aim of the study was to understand nutritional assessment and management, which is an assessment of HCP knowledge and behaviour, the patients were able to collaborate data collected from the HCPs.

### 2.2. Context 

Data collection was loosely underpinned by principles of phenomenology. Phenomenology in its broadest sense is concerned with consciousness and the subjective view of experience [17] In this case, we were looking at professionals’ and patients’ lived experiences of the identification and treatment of malnutrition in LMICs. The phenomena under investigation were nutritional screening, assessment and supportive interventions separating the ideal from the actual reality. The qualitative semi-structured interviews and focus groups aimed to formulate a baseline understanding of the participants’ lived experience. It was envisaged that this method would enable a greater insight regarding the phenomena under investigation. The interviewers were seeking an understanding of the identification and treatment of malnutrition in LMICs to inform the development of a feasibility study in Zambia, Ghana, the Philippines and India [18]. 

### 2.3. Data Collection 

The semi-structured interviews and focus groups were conducted either face-to-face or via video conferencing and followed an interview schedule, which was adapted for focus groups (Supplementary Material S1). Each participant was either interviewed once or took part in one focus group. In one of the patient interviews, a family member was present with patient permission. Interviews ranged from 10 to 45 min and the focus group lasted for one hour. Interviewers followed a standard operating procedure developed by S.T.B. This interviewer was a dietitian with experience in conducting qualitative interviews and, as part of the research team, did not have a professional relationship with the interviewees. The other interviewers were surgeons (A.V. and C.A.) and used the same procedures for the interviews. They were known in a professional capability by the interviewees. All the interviewers were female with an interest in the place of nutrition in surgical oncology. All the interviewees were aware that the interviewers had an interest in nutrition research. Interviews were conducted in English, apart from those carried out with patients in the Philippines, which were conducted in Filipino. Interviews were digitally recorded, and transcribed. Transcripts were translated into English by native speakers where necessary, but not returned to participants for comment. Data were collected until saturation was reached when no new themes or topics were being identified from the interviews. This was achieved as the interviews were translated, transcribed and analysed in parallel to data collection. 

### 2.4. Data Analysis

Data were managed using NVivo Version 12 (QSR International Pty Ltd., Doncaster, VIC, Australia) and analysed thematically using the six-step process from Braun and Clarke [19]. These steps are familiarisation, generating codes, developing themes, reviewing themes, defining themes and the final analysis. One researcher (A.M.S) coded the data. Another researcher (S.T.B) independently reviewed the codes. Codes and themes were discussed and agreed upon between A.M.S and S.T.B. Whilst the researchers were open to themes from the data, the research was aiming to identify barriers and facilitators to nutritional assessment and management on surgical cancer wards in LMICs. Quotations were taken from the transcripts as example data to support the themes being derived from the content. Even where the interviews were carried in English, as English was not the participant’s first language, quotations were edited to increase comprehension. However, care was taken to not alter the meaning or sense of the data. Quotations are identified by the first letter of the country and whether the individual was an HCP or a patient. 

Ethical approval was obtained from the University of Edinburgh Ethics Committee and where necessary, local ethical approvals were sought in the individual countries. All participants gave informed consent but did not comment on the findings. 

### 2.5. Rigour

Rigour was introduced by having two researchers discuss and agree on the codes and themes. In addition, the consolidated Criteria for Reporting Qualitative Research (COREQ) checklist was used [20]. 

## 3. Results

### 3.1. Sample Details 

In total, 34 participants were included in this qualitative study. There were 10 patients from the Philippines. In addition, there were 24 HCPs: 16 were interviewed and 8 were involved in a focus group. HCPs were from Ghana, India, the Philippines and Zambia. There were two nurses, ten dietitians or nutritionists, three oncologists, one study coordinator and eight surgeons.

### 3.2. Themes

**Figure 1:**
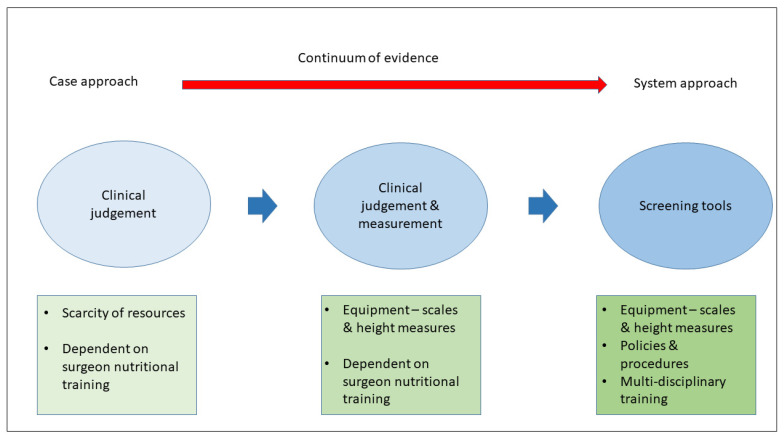
Nutritional assessment in LMICs.

Four themes were identified in the data. These were the experience of participants of (1) nutritional assessment, (2) support, (3) the barriers and (4) facilitators of nutritional management. An overview of the practices in different countries is provided in Figure 1.

#### 3.2.1. Nutritional Assessment

The participants reported a variety of practices relating to nutritional assessment (see Figure 1). At one end of the spectrum, there was no systematic evaluation of nutritional status and assessment was based on observing whether or not the patient looked malnourished. A surgeon from Zambia stated that assessing patients with cancer “you use clinical judgement to [see if] this one looks wasted” (Z-HCP2). This judgement could be supported by a history of weight loss reported by patients and if the patients lived in an economically deprived area. In this scenario, patients would rarely be weighed, as the surgeon commented, “Weighing is neither here nor there” (Z-HCP2). 

A similar situation existed in Ghana. A surgeon commented that he would use height and weight to assess nutritional status. However, as these were not taken routinely, he also relied on observing patients, referring any who looked wasted to the nutritionist. Nutritionists in Ghana commented that although most patients with cancer would be malnourished, there was no established pathway for treating malnutrition and referring to them was at the discretion of the doctor. In contrast, surgeons from India reported routinely using the objective measures of weight and height to assess nutritional status. One commented that weight, height and body mass index (BMI) would be recorded in the nursing notes and the anaesthesiologist would take these measurements again. Having these measures consistently taken by two HCPs would ensure that patients did not slip through the gaps. For these surgeons, scales and height measures were readily available “height measures and scales are available on the wards” (I-HCP1). However, calibration of the scales could be a problem, as one surgeon said, “They are not calibrated” (I-HCP1). Although this was not always the case, another commented that in his hospital, there was a cycle for scales to be calibrated “all instruments have to be calibrated in a specific period of time… in 6 months or 1 year” (I-HCP2).

Although surgeons from India reported using the objective measures of weight and height to assess nutritional status, screening tools were not normally used in their hospitals. As one surgeon commented “people are just not aware that there are these screening tools…people think that if albumin is normal and BMI is ok then nutrition is ok…they are not aware that with a normal BMI and albumin patients can be malnourished” (I-HCP1). 

These experiences can be compared to a systematic approach to assessing nutritional status reported by HCPs in the Philippines. The initial assessment would occur when patients attended as outpatients: “Even upon the first consult of the patient, we assess the patient” (P-HCP4). The doctors reported using a methodical approach. They would ask about weight loss, “We ask…about the weight loss, first directly” (P-HCP2). If the patients could not answer directly, the doctors would probe further using ”surrogate parameters like if their clothes become loose, or if other people have been noticing that they are losing weight” (P-HCP1). They would also ask about oral intake and the patient’s appetite. 

Further nutritional assessment of patients occurred on admission using a screening tool. As one said, “for our screening tool…we use the Nutritional Risk Assessment tool” (P-HCP4). Part of that screening includes measuring a patient’s weight and height. Thus, subjective assessment would be combined with making use “of the objective method, when we get their height, weight” commented a dietitian (P-HCP5). From weight and height, dietitians would calculate BMI.

#### 3.2.2. Nutritional Interventions

The purpose of nutritional assessment is to identify and treat patients who are undernourished. If the person was an outpatient, doctors in the Philippines could refer them to the nutritionist for advice. In other countries, the surgeon might advise the patients themselves. As one surgeon in India commented, he would prescribe supplements and a multivitamin. However, patients would have to buy the supplements. Therefore, surgeons might give advice about what foods to eat. As one surgeon in Zambia commented, patients might be advised to eat more nutritious foods that they could buy for the same cost; for example, “get eggs or milk instead of cornflakes” (Z-HCP2). A surgeon in India recommended increasing protein from food, he advised “those who can take non-vegetarian diet. I encourage them to have egg whites—6–8 in a day. If a patient cannot, we advise them to have cottage cheese, 100 to 200 g cottage cheese”. For those who cannot take solid food, he advised “soya-based protein, two to three scoops in milk” (I-HCP2). 

Once admitted, treatment of malnutrition seemed to be easier, with patients being given oral supplements. This could be readymade supplements from the pharmacy, which HCPs in the Philippines said were available to patients, “if they are admitted…it’s actually free” (P-HCP8). Alternatively, nutritionists could prepare nutritional supplements in the hospital kitchen. This occurred in Ghana, as commented on by a surgeon and dietitian. The dietitian said that they “do not have prepacked therapeutic food, but fortunately as nutritionists we are trained to prepare the local foods” (G-HCP3).

Healthcare professionals commented that parenteral nutrition was very expensive and rarely used. However, HCPs in the Philippines said it was occasionally used and surgeons in Zambia said they might have it if there had been donations. 

#### 3.2.3. Barriers to Nutritional Care 

There were a range of barriers related to the assessment and treatment of malnutrition. First, there could be no systematic treatment pathway or protocol of screening patients for malnutrition, as commented on by HCPs in Zambia and Ghana. However, even when a pathway existed for identification of malnutrition such as in the Philippines, there was a problem of whether this happened in practice. A surgeon said that it was the junior resident’s job to screen the patients using the Nutrition Risk Score screening tool, but “they never complete them, maybe they complete in the first week, then they forget about it” (P-HCP11). However, other HCPs talked about the nurses completing the screening tool. A dietitian said, “the screening tool basically is a task for the nurses…supposedly upon admission, the patients are screened to determine who are nutritionally at risk or not”, and that they had provided training for the nurses: “we conducted a series of seminars” (P-HCP5).

A recently qualified nurse collaborated this saying that she had completed screening tools during her undergraduate training. Although, there seemed to be other issues with the nurses completing the tools now. The dietitian said, “I just lost track if the nurses do this…because of the pandemic” (P-HCP5). The nurse commented on a different problem “since we use [Electronic medical records], most of our charts are computerised. Then there’s no feature in [Electronic medical records] that the old chart is included there so mostly it’s just words. Charting as if it’s a journal entry” (P-HCP6). This would mean that the nurses would not have the questions as a prompt, and it would be easier to miss doing the assessment. 

Weighing patients is a key aspect of assessing nutritional status. As noted above, this was not routine practice for surgeons in Zambia and Ghana. A nutritionist in Ghana commented, “You cannot do much without weighing the patient for energy requirements and assessment” (G-HCP2). However, they were hindered in this, as they did not have scales readily available. Whereas, weighing patients was usual for HCPs interviewed from India and the Philippines. Interviews with patients in the Philippines confirmed that weighing of patients was happening routinely—only one person who could not stand had not been weighed. 

Availability of food in hospital is a crucial element to maintain the nutritional status of inpatients. In Ghana, both a surgeon and dietitians commented that the food supply from the hospital kitchen was precarious. The dietitians said that many patients came from out of town and needed to rely on food from the hospital kitchen, but it was not reliable. In contrast, HCPs from the Philippines said that patients received a ration of food daily from the kitchen and patients or relatives would generally buy food to supplement it. Patients had a range of views on the food from “It’s fine…it’s enough” (P-Pt6) to “it shouldn’t be bland. Because people like us patients, sometimes we have poor appetite that’s why we eat so little” (P-Pt9).

If patients required oral supplementation in addition to food, there were various problems with patients accessing them. Dietitians in Ghana did not have access to readymade supplements but would make them themselves in the hospital kitchen. However, one dietitian commented, “We do not always have the food and ingredients for the recipes.” In addition, they did not have access to kitchen scales to weigh ingredients and had to rely on handy measures. HCPs in the Philippines reported that readymade supplements were freely available in their hospital. However, the pharmacy would buy a three-month supply and would not be able to purchase more in between so occasionally stocks would run out. Patients reported a variable experience of having supplements. One patient reported, “I have lost my appetite” and when asked if they had been given supplements said, “not yet” (P-Pt7). Whereas another patient who had lost their appetite said they had been given, “milk called Boost Optimum that was prescribed to me” (P-Pt9).

In all countries, there was a particular problem for patients accessing oral supplements outside the hospital, as they need to buy them, and the cost was prohibitive for most. Nevertheless, patients in the Philippines mentioned being given cartons of oral supplements. As one said, “I was given [oral supplement] but I left it at our place” (P-Pt1) and another commented, “we were visited by…officials. They gave out [oral supplement] to the elderly” (P-Pt10). However, it does not seem to have been either a consistent supply or consistently taken by the patients. 

There were also barriers for patients accessing appropriate advice as outpatients as access to nutritionists was limited for patients in the community. HCPs in the Philippines said that patients could be referred to a dietitian as an outpatient. However, patients could have to travel long distances to come to the hospital. They would not want to come only for nutritional assessment and monitoring. If they were at the hospital, patients did not want to wait to see the dietitian if the queue was long.

#### 3.2.4. Facilitators for Nutritional Care 

Although there were many barriers, there were circumstances that could be leveraged to facilitate good nutritional care, such as the training and level of knowledge of the HCPs. For example, the dietitians in Ghana were aware of how to undertake assessments and prepare supplements. They would do so if the resources were in place. They were also keen to research their practice to improve outcomes for patients; a nutritionist said, “I would love to do more research on what we are preparing and if it really meets the standards for protein, carbohydrates and whatever” (G-HCP3). HCPs in other countries also demonstrated their knowledge of nutritional assessment. The surgeons in India discussed the importance of weighing patients and HCPs in India and the Philippines commented on the value and use of screening tools. Therefore, although physical resources could be scarce, intellectual and knowledge resources were available, without which nutritional assessment is not feasible. 

The other asset that some HCPs reported were the systems that were in place in their hospitals, which facilitated nutritional care. For example, as noted above, a surgeon in India commented that there were mechanisms to ensure that patients were weighed, as patients were weighed both on admission by the nursing staff and by the anaesthesiologist. In the Philippines, there was a system for filling out the screening tools. Although there were current problems with it being carried out, the training and infrastructure were in place to be built on. Having systems and protocols in place is a more efficient way to ensure that patients are not missed and that the scarce resources are used appropriately.

## 4. Discussion

In this study, we explored nutritional assessment and support on surgical cancer wards in LMICs. We found that the knowledge of nutritional assessment amongst HCPs was high. However, there was a continuum of care moving from individual assessment using only clinical judgement, to subjective assessment and objective assessment, and finally, to a system approach employing screening tools.

The continuum of care from clinical judgment to a systems approach seemed to be fuelled by availability of resources. Clinicians only used clinical judgement if they did not have access to weighing scales, and as physical resources increased, there was also an increase in systems for assessing nutritional status, with hospitals having protocols for weighing patients and the use of screening tools. In our study, only HCPs in the Philippines working in a University hospital setting reported that they had a system in place for using screening tools. Screening a patient’s nutritional status on admission is an effective and efficient way of identifying malnourished patients [21]. Training and systems are required to embed screening tools into routine practice with a view to forming an institutional culture which prioritises nutritional care [22]. Training is an important aspect of ensuring that patients with cancer have adequate nutritional information [23]. This has cost and resource implications. However, this upfront cost needs to be balanced by the costs of not providing adequate nutritional screening and support. It is known that malnutrition in surgical patients in LMICs is high and is associated with increased levels of morbidity and mortality [24,25,26]. This has consequences for costs and the overall length of hospital stays. In addition, screening for malnutrition ensures that nutritional support goes to the patients who need it most, which is another consideration in contexts where resources are scarce. Moreover, demonstrating to service providers and funders that nutritional management is a cost-effective measure may lead to more organisational change within healthcare structures and targeted allocation of resources. 

Part of the screening process is finding out whether patients have lost weight in preceding months, and in some LMICs people would not routinely measure their weight, as discussed by HCPs in this study. In these circumstances, HCPs use surrogate measures to assess patients’ weight loss, such as whether their clothes have become looser. Researchers have suggested that screening tools are modified to incorporate such measures [27]. This would ensure that validated measures are available, which are suitable in these circumstances. 

Food first is recommended for nutritional support in many countries [28]. Therefore, hospital catering systems have an important role to play in maintaining nutritional status and treating malnutrition. However, hospital catering systems are often unable to provide meals that will meet the nutritional needs of all patients, particularly those with cancer who have the most to gain from targeted interventions [21]. There is a need to increase awareness of the importance of providing adequate nutrition and the important role that the catering system has in this [29,30,31]. Often, relatives needed to supplement meals that patients received whilst in hospital. It has been suggested that family members are given education and adequate cooking facilities to ensure the provision of optimal nutrition to patients [32]. 

A strength of the current study is that HCPs from four different countries were involved, providing a broad picture of nutritional assessment and support across a range of LMICs. A further strength is that patients were also interviewed in the Philippines, which corroborated the observations of the HCPs and provided the unique patient perspective on the topic. They provided triangulation of data, where data is drawn from different sources, and as such were playing a supportive, but not essential role [16]. Therefore, although it would have been beneficial to have patients based in other countries, this was not imperative. In addition, qualitative work allows the understanding of the nuances around people’s experiences of nutritional care in a real-world environment. A potential limitation is that the sample was taken from tertiary centres and there is a chance of significant variations between hospitals within each country. However, the more important consideration is whether variation in the resources between hospitals mirror our findings. That is, if HCPs are knowledgeable about nutrition, but resources are limited, is a case-based rather than a systems approach adopted? This would be an interesting point for further investigation.

The study has identified variability in the practices of HCPs and resources in LMICs. Further work needs to be carried out in addressing malnutrition for cancer patients in these settings. However, the identification of low resources and high costs could lead to the development of local products and protocols. Some researchers have investigated the use of cost-effective, locally manufactured supplements [33,34]. This work could be further developed. In addition, guidelines suitable for LMICs are required. Those published by high-income countries highlight the best evidence but do not consider the financial and supply chain factors faced by most LMICs. Protocols for LMICs need to take into account both the best evidence and the local situation.

## 5. Conclusions

In conclusion, nutritional management of surgical cancer patients in LMICs could be improved by moving from individual case management to a system-based approach, where nutrition support management is integrated into routine hospital policies and procedures. The challenges of implementation in low-income settings should not be underestimated. The development of a more robust evidence base relating to the effectiveness of nutritional supplementation for patients having surgery for cancer is required to influence policies and justify increased resource allocation within healthcare systems in LMICs. 

## Data Availability

The data are not publicly available due to participant confidentiality.

## Supplementary Materials

The following are available online at https://www.mdpi.com/article/10.3390/nu14040863/s1, Interview Schedule (Supplementary Material S1).
