# Supplementary material for: A Qualitative Exploration of Nutrition Screening, Assessment and Oral Support Used in Patients Undergoing Cancer Surgery in Low- and Middle-Income Countries
Source: Nutrients. 2022 Feb 18;14(4):863. doi: 10.3390/nu14040863 (PMC8876193; doi:10.3390/nu14040863)
Supplement: Supplementary file 1 [file nutrients-14-00863-s001.zip › nutrients-1582261-supplementary.pdf]

CRANE

a trial by the

NIHR Global Health Research Unit on Global Surgery

Inclusion Criteria

- Over 18 years of age (or the age of majority for the country);
- Patients who have or are undergoing planned elective surgery for cancer;
- Participants must be able and willing to provide written informed consent (signature or a fingerprint).

Interview Schedule for patients

Participant number:
Country:
Age<br><br>Male Female<br><br>Date of surgery<br><br>Operation<br><br>Type of cancer
Interview date:
Interview start time:
Interview end time:

Please record the interview and translate if required. The translation can be verbal or recorded. Please email the anonymised recorded interviews to sorrel.burden@manchester.ac.uk or transfer anonymised recordings via "Whatsapp"

Schedule

We are interested in asking you a few questions about what you are eating before your operation.

1. What have you had to eat today?

2. How has your appetite been over the last week?

3. Have you been weighed or has any one talked to you about your weight?

4. Have you lost any weight?

5. If you have lost weight when did you first notice this weight loss?

6. If you were to have some food before your operation what would you be able to manage?

8. Could you take a supplement in the form of a paste or a liquid?

9. What foods do you most feel like eating now?

10. What foods were you managing to eat when you were at home?

11. How has the surgery and cancer effected you’re eating habits?

12. How do you obtain your meals in hospital?

Home/Vendors/Hospital

13. What is the perception of the food you receive from the hospital?

14. Have you ever taken any supplements in the form of paste or liquid?

15. If “yes” please specify……

16. Do you have anything else to add about food in hospital or before your illness?

Thank you

Focus group/Interview Schedule for Healthcare Professionals

We are interested to find out your views on nutritional assessment and also foods or drinks that are available to help patients before surgery to build them up and improve their nutritional status.

During the next hour or so we are going to ask you some questions about nutritional assessment also foods that can be used to build people up before surgery.

First of all, I would like to show you some screening tools used in other hospitals and see what you think.

Would any of these tools be helpful in your hospital?

What do you like about the tools?

What do you not like about the tools?

How do you assess if someone has lost weight or is undernourished at the moment?

When would it be noticed if a patient was losing weight or undernourished at your hospital?

Outpatients
On admission
At any point before and after surgery

Are there any questions or things that alert you to weight loss or undernutrition used in your hospital?

Clothes becoming loose
Moving belt up a few notches

Do patients who have lost weight or who are undernourished get any special foods or products?

If so, what do you use?

Do you have any ideas of what would work well in your hospital to help build people up?

Do you think there would be any problems giving something to help build people up?
